# Diagnostic and Therapeutic Implications of the SUMOylation Pathway in Acute Myeloid Leukemia

**DOI:** 10.3390/cancers17040631

**Published:** 2025-02-13

**Authors:** Elena Chatzikalil, Konstantinos Arvanitakis, Filippos Filippatos, Panagiotis T. Diamantopoulos, Theocharis Koufakis, Elena E. Solomou

**Affiliations:** 1 First Department of Pediatrics, National and Kapodistrian University of Athens Medical School, 11527 Athens, Greece; filippat@med.uoa.gr; 2 “Aghia Sofia” Children’s Hospital ERN-PeadCan Center, 11527 Athens, Greece; 3 Division of Gastroenterology and Hepatology, First Department of Internal Medicine, AHEPA University Hospital, Aristotle University of Thessaloniki, St. Kiriakidi 1, 54636 Thessaloniki, Greece; arvanitak@auth.gr; 4 Basic and Translational Research Unit, Special Unit for Biomedical Research and Education, School of Medicine, Faculty of Health Sciences, Aristotle University of Thessaloniki, 54636 Thessaloniki, Greece; 5 First Department of Internal Medicine, National and Kapodistrian University of Athens Medical School, 11527 Athens, Greece; pandiamantopoulos@gmail.com; 6 Second Propaedeutic Department of Internal Medicine, Hippokration General Hospital, Aristotle University of Thessaloniki, 54642 Thessaloniki, Greece; thkoyfak@auth.gr; 7 Department of Internal Medicine, University of Patras Medical School, 26500 Rion, Greece; esolomou@upatras.gr

**Keywords:** SUMOylation, acute myeloid leukemia, chemotherapy, resistance, epigenetic biomarker

## Abstract

Epigenetics encompasses heritable and stable changes in gene expression caused by external chromosomal modifications, without altering the underlying DNA sequence. Epigenetic modifications, established during early development and maintained through successive cell divisions, play a critical role in regulating gene expression. Post-translational modifications (PTMs) are a key aspect of epigenetics and are essential for modulating protein functionality, as well as regulatory cellular processes, including proliferation, differentiation, metabolic pathways, and tumorigenic events. Among these, the small ubiquitin-related modifier (SUMOylation) system is a reversible PTM mechanism that alters target protein interaction surfaces through covalent binding to lysine residues, thereby influencing protein structure and function. Acute myeloid leukemia (AML) is a highly aggressive malignancy characterized by the clonal expansion of primitive hematopoietic stem cells of the myeloid lineage in the bone marrow. Despite recent advancements in therapeutic strategies and an improved understanding of leukemogenic pathways, patient outcomes remain poor, particularly in elderly populations. Consequently, efforts have focused on developing novel agents, including co-targeting specific mutations or integrating targeted therapies into combinatorial chemotherapeutic regimens. Emerging evidence suggests that SUMOylation plays a significant role in AML pathogenesis and treatment response, representing a promising therapeutic target for advanced disease cases. This review provides a brief analysis of the functional role of the SUMOylation system in AML and highlights its potential as a therapeutic target. We also discuss current knowledge gaps and propose directions for future research to advance precision medicine approaches for AML treatment.

## Simple Summary

Small ubiquitin-like modifier proteins represent a highly conserved family of post-translational modification proteins, which play multiple roles in regulatory cellular processes, concerning both healthy and pathological conditions. Recent in vitro studies and phase I clinical trials have highlighted the dysregulation of SUMOylation in various hematologic malignancies, including acute myeloid leukemia, where it is implicated in leukemogenesis and the modulation of cancer cell chemosensitivity. In this literature review, we aim to summarize current evidence on the role of SUMOylation in AML development and progression, emphasizing the scientific rationale for targeting this pathway to enhance patient survival outcomes.

## 1. Introduction

Epigenetics are heritable changes in gene expression that modify the translation of a genotype into a specific phenotype, without changing the genetic material [1]. Epigenetic modifications concern both types of nucleic acids and belong to the most expanding fields of human biology, attracting increased research interest, especially after the introduction of nucleic acid deep-sequencing technologies [2]. Epigenetic modifications can be induced by various epidemiological and environmental factors, including alterations in DNA methylation profile, regulation of chromatin by histones, and small non-coding microRNA molecules (miRNAs), and are, in most cases, reversible and rarely passed to the next generations, despite persisting through multiple cycles of DNA replication [3,4]. PTMs represent gradually developed alterations in protein structure and chemical properties, consisting of proteolytic cleavage and covalent addition of a functional protein group (acetyl, phosphoryl, glycosyl, or methyl) to at least one amino acid [5]. PTMs, namely phosphorylation, glycosylation, ubiquitination, nitrosylation, methylation, acetylation, lipidation, and proteolysis, regulate a great variety of protein functions, including life cycle, interactions with cellular molecules and co-factors (proteins, nucleic acids, lipids, cell matrix), secretory trafficking pathways, receptor activation, protein solubility, and folding and processing into the endoplasmic reticulum [6,7,8]. SUMOylation is a PTM that presents multiple emerging roles at a cellular level, regulating transcription, nuclear integrity, proliferation, senescence, lineage commitment, and stemness, being involved in a great variety of pathological conditions [9,10]. During the last decades, multiple precision oncology protocols have been developed based on the efforts to adopt novel targets as a part of combinatorial schemas for highly metastatic cancer types [11,12,13]. In this context, the role of SUMOylation in neoplastic disease has been investigated, and pathological SUMOylation of distinct protein products or holistic dysregulation of the SUMO signaling pathway has been identified in numerous neoplasms [14]. Moreover, SUMOylation pharmaceutical targeting has been hypothesized to potentially accomplish promising clinical responses in many types of malignancies [15].

AML is a myeloid malignancy, characterized by the dysregulated proliferation and differentiation of hematopoietic cells of the myeloid lineage, within the bone marrow [16]. AML is characterized by increased rates of refractoriness, relapse, and mortality, with the 5-year survival rates almost reaching 30% a decade ago [17]. During the past ten years, the available therapeutic choices, which had remained stagnant for decades, have constantly changed, currently including Bcl-2 inhibitors (venetoclax), FLT3 inhibitors (midostaurin), and IDH1/2 inhibitors (ivosidenib) [17,18]. For patients with advanced AML, the prognosis remains poor, and intensive induction chemotherapy is followed by a post-remission consolidation treatment including allogeneic stem cell transplantation [17], while other AML subtypes, for example, acute promyelocytic leukemia (APL), show a favorable prognosis when treated with the established chemotherapeutic combination schemas [18,19,20,21]. Recent progress in the fields of personalized medicine aiming to offer therapeutic choices, even in the most advanced AML cases, has led to the identification of several molecules that are currently considered major contributors to leukemia tumorigenesis and progression, suggesting new targeted therapies for refractory AML [22,23]. Aberrant epigenetics in AML were first described in 1987, with the introduction of altered DNA methylation in the 5′ regulatory region of the calcitonin gene in leukemia cell lines [24]. Follow-up studies reported that 5-methylcytosine expression is not significantly different in newly diagnosed acute leukemias, while it presents significantly decreased expression levels at relapse, suggesting that hypomethylation is not involved in the initiating steps of leukemogenesis, but it is rather considered as an additional “hit”, leading to chemoresistance [25]. After these primary steps, continuous investigation, and clinical studies on the application of personalized therapeutic approaches, based on epigenetic alterations, patient-specific and disease-specific risk stratification factors have been proposed and promising combinations of targeted medication have been suggested.

Herein, we analyze the current evidence regarding SUMO epigenetic alterations in AML, the potential role of these changes in leukemogenesis and disease progression, and the promising utility of this knowledge in designing more effective targeted therapeutic strategies.

## 2. SUMOylation Cascade and Its Tumorigenic Potential

### 2.1. Isoforms, Structure and Signaling Mechanisms

SUMO represents a family of highly conserved isoforms of ubiquitin-like post-translational modification proteins (namely SUMO1, 2, 3, 4, and 5) [26]. SUMO1, 2, and 3 are widely expressed in all tissues throughout the human body and represent the majority of SUMO modifications, while SUMO4 and SUMO5 show restricted expression in specific human tissues [27,28,29]. SUMO2 and SUMO3 are 97% sequence identical, being both 50% sequence homologous with SUMO1, and the expression levels of SUMO isoforms (SUMOs) differentiate between the distinct developmental stages [30]. SUMOs bind either to one (monoSUMOylation) or numerous (multiSUMOylation) lysine side chains, while SUMO molecules also have the ability to SUMOylate themselves (polySUMOylation), creating polymeric SUMO chains [31,32]. Being compared to other well-studied epigenetic pathways, SUMO presents high similarity, in terms of structure and functionality, with the ubiquitin epigenetic system [33]: the molecular characteristics of SUMO are similar to their ubiquitin system’s counterparts, with the “ββαββαβ” structure being among the main structural elements and the diglycine (GG) motif being necessary for isopeptide bond formation [34,35].

SUMO signaling cascade consists of the conjugation of the C-terminal carboxylase to the ε-amino lysine residues, namely SUMO E1-activating, E2-conjugating, and E3 ligase, by an isopeptide bond [36,37]. SUMO activation is a result of its binding to the reactive cysteine residue of a heterodimeric SUMO E1-activating protein (Aos1/Sae1-Uba2/Sae2) and its subsequent transfer to the reactive cysteine residue of an E2 SUMO-conjugating protein (Ubc9/Ube2i) [37,38]. E3 ligase enzymes, which enhance SUMO transportation from the E2 to lysine residues, are not yet fully characterized [39]. Between the most well-studied ones are RanBP2, which allosterically activates Ubc9, SP-RING domain, which facilitates Ubc9 binding to its substrates, ZNF451, which elongates SUMO2/3 chains, and the transcriptional co-regulators termed “protein inhibitors of activated STAT (PIAS)”, consisting of seven distinct enzymes: PIAS1, PIAS2a (PIASxα), PIAS2b (PIASxβ), PIAS3, PIAS3L, PIAS4 (PIASy), and PIASyE6− [40,41,42,43]. Sentrin-specific proteases (SENP1, 2, 3, 5, 6, 7, and 8) are responsible for the maturation and deSUMOylation of the SUMO cascade [41]. SUMO undergoes a continuous cross-talk with several PTMs, mainly phosphorylation and methylation, which may up- or downregulate SUMOylation processes [44,45,46].

**Figure 1:**
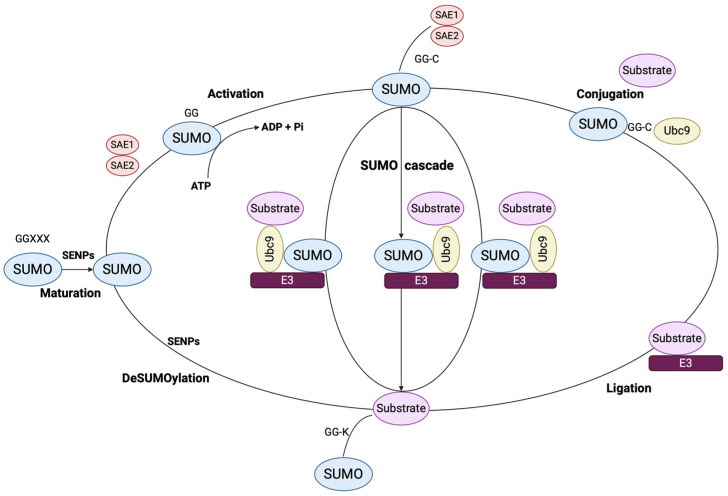
An overview of the SUMOylation pathway. SUMO peptide is cleaved at the covalent C-terminal region by SENPs into a mature form with a GG motif. SAE1 and SAE2 activate SUMOylation via their interaction with the terminal GG of a mature SUMO protein, while ATP is present. SUMO protein is subsequently transferred to the reactive cysteine of a Ubc9, which continues transferring SUMO to a substrate. DeSUMOylation is catalyzed by SENPs and SUMO signaling is reversed. [GG: glycine–glycine; SAE1/2: E1/2 SUMO-activating enzymes; SENPs: sentrin-specific proteases; SUMO: small ubiquitin-related modifier; Ubc9: E2 SUMO-conjugating protein]. (The image was created using BioRender software version 04, License #QF27W7DXJN).

An overview of the SUMOylation signaling cascade is illustrated in Figure 1.

### 2.2. Implications in Carcinogenesis

Considering the crucial role of the SUMO pathway in a variety of cellular processes, for example, cell growth and differentiation, it could easily be expected that dysregulation of SUMOylation may promote tumorigenic processes, including proliferative and apoptotic pathways [47,48]. Dysregulation of the SUMO signaling cascade has lately been reported to present potential prognostic implications in cancer patients, with increased expression levels of SUMO machinery components (quantified via genome-wide analyses and mass spectrometry-based methods), being positively correlated with histological grade, stage, metastatic capacity, and overall survival rates (with several exceptions, e.g., SENP2 and PIAS) [49,50,51,52]. The pathologically altered expression of SUMOylation components is, in some cases, already detectable at the mRNA level; a characteristic example of this type of dysregulation is the induced expression of SENP6 in hepatocellular carcinoma caused by the hypomethylation of SENP6 domains, which is highly correlated with disease progression and worse prognosis [53,54]. At the post-transcriptional level, a great variety of miRNAs are involved in SUMO regulation, and their expression is a potential indicator of tumor progression in various types of malignancies [55,56,57]. For instance, in vitro downregulation of miR-145 has been associated with increased SENP1 expression levels in prostate cancer tissue samples, while enhanced expression of miR-145 has been proven to result in cell cycle arrest by blocking SENP1 [58]. Moreover, miRNA-9 and miRNA-181a are recently defined inhibitors of PIAS3 in breast cancer with high IL-6 expression, inducing the expansion of immature myeloid-derived suppressor cells, leading to suppression of T-cell immunity [59].

Recent investigation has shed light on SUMO implications in oncogenic signaling pathways and in distinct tumor microenvironment (TME) components, which hold great clinical significance from a prognostic and theranostic perspective in solid and hematologic malignancies [60,61,62]. NF-κB, TGF-β, and JAK/STAT signaling cascades are considered to be promoted or antagonized by using SUMO targeting therapeutic applications [63,64,65]. Regarding NF-κB, it is the most well-studied linking factor between SUMOylation and inflammatory TME; simultaneous SUMOylation of mesencephalic astrocyte-derived neurotrophic factor (MANF) enhances the translocation of MANF to the nucleus, blocking both NF-κB/Snail cascade and epithelial mesenchymal transition in liver tumors [66]. Moreover, TRIM60 SUMO alterations in K329 and K562 inhibit MAPK/NF-κB activation by disrupting TRAF6/TAK1 complex formation, dysregulating innate immune response [67]. SUMOylation is also dysregulated in hypoxic TME conditions, enhancing proliferation, invasion, metastasis, and tumor cells’ chemoresistance [68,69,70]. Furthermore, given the established mechanistic role of SUMO components in metabolic dysregulation (specifically in insulin exocytosis amplification, NADPH generation, and glutathione reduction), the role of SENP1 has been the main area of recent investigation on SUMO implications in the NEMO pathway, which has revealed its crucial role as a regulator of metabolic reprogramming of TME formation [71,72]. A well-designed in vivo investigation is warranted in order to verify these findings and provide clinically applicable evidence for the role of SUMOylation in cancer diagnosis and prognosis.

### 2.3. SUMOylation as a Part of Epigenetic Modification Patterns of AML

During the last ten years, evidence regarding the molecular pathogenetic mechanisms of AML has dramatically increased, mainly due to the wide use of next-generation sequencing and whole genome sequencing technologies [17,18]. AML is a hematologic malignancy with significant mutational load and genetic heterogeneity, a fact which re-enforces the investigation of novel therapeutic targets for patients with advanced disease [22,73]. Epigenetic studies have revealed that dysregulation of DNA methylation is associated with clonal expansion and that DNA epigenetic changes act synergistically in developing the AML phenotype. Based on these observations, several authors suggest that epigenetic heterogeneity, rather than genetic profiling, could better explain AML pathogenesis [74]. In more detail, DNA methyltransferases participate in stem cell self-renewal and differentiation, leading to dysregulation of normal hematopoiesis, which allows the use of DNA methylation patterns for risk and outcome stratification of patients [75]. Histone modifications are tightly interacting with DNA methylation processes, synergistically leading to clonal evolution of hematopoietic stem cells, which has been identified in preleukemic stem cells via deep sequencing technologies, with DNMT3A being the most common mutation identified [76]. These groundbreaking findings turned research interest to studying epigenetic alterations in AML as key events during the development of the disease. In this context and given that SUMO components are amongst the first studied PTMs in some subtypes of AML (APL AML), studying the SUMOylation PTMs in AML patients could be an area of further research, potentially offering new therapeutic choices in patients with advanced or refractory disease.

## 3. Role of the SUMO Pathway in AML: Novel Insights in Leukemogenesis and Response to Treatment

### 3.1. SUMO in Acute Promyelocytic Leukemia (APL)

APL stands among the most successful cases of targeted therapy induction in cancer treatment, showing complete remission in more than 80–90% of patients [77]. Complete remission can be achieved with a chemotherapy-free approach with the use of all trans retinoic acid (ATRA) and arsenic trioxide (ATO). A combination of ATRA and ATO with concomitant use of gemtuzumab ozogamicin (GO) or idarubicin is used for high-risk patients [77,78]. Given the very high rates of complete remission, the introduction of implementing more efficient treatments as well as the affordability of further investigation are challenging. However, there is a small percentage of patients with relapsed APL and high early death rates (EDR), with coagulopathy and hemorrhage being considered as the main EDR causes [79]. The lack of availability of therapeutic choices for these cases currently remains the only obstacle in APL treatment. Ongoing clinical trials are investigating novel therapeutic options for these patients, including per os arsenic formulations, as well as retinoic acid agonist tamibarotene, mainly in cases of relapse or advanced disease [80,81,82]. Interestingly, investigation of PTMs has revealed that the SUMO signaling pathway is a major regulator of APL chemosensitivity, being associated with specific chromosomal translocations involving the retinoic acid receptor α (RARα) gene (RARA) on chromosome 17 and leading to the formation of the promyelocytic leukemia retinoic acid receptor α protein (PML-RARα) [83,84]. PML-RARα is amongst the first characterized SUMOylated proteins and a good substrate for SUMOylation [85], negatively affecting intracellular nuclear structures termed nuclear bodies (NBs), which were recently demonstrated as main sites of SUMOylation [86,87,88,89]. The SUMO effect on PML-RARa results in the inhibition of apoptosis and proteasome-mediated de-repression of myeloid differentiation via cycles of polySUMOylation and polyubiquitination [86,87,88,89]. Considering the fact that PML-RARα is the first proposed protein model for the study of SUMOylation in AML, further exploration of PML NB-enhanced SUMOylation may give answers for some groups of patients with APL subtypes that are currently untreatable [85].

Additionally, given that PML is not only an important component of the outer surface of the NB spheres but also regulates nuclear NB transportation, PML SUMOylation is considered responsible for NB shaping, sequestering, modifications, formation, and NB protein interactions [90]. In more detail, PML in NBs displays a SUMO interacting motif based on the intermolecular interplay between the SUMOylated PML protein and the SIM protein, located on the PML C-terminus [91]. Recent evidence not only highlights the crucial role of the SUMO pathway in NB formation but also proposes a potential mechanism for NB-mediated stress-induced SUMOylation [92]. Specifically, NB formation consists of an initial oxidative stress-induced PML multimerization and a polarized non-covalent interaction between the SUMO pathway and SIM protein, which results in the recruitment of the interacting paired proteins [92]. SUMO dysregulation results in impaired NB formation, while PML SIM may tether SUMOylated peptides onto NBs [92]. A characteristic example of this observation is K160, a primary SUMO binding site on PML protein, whose modification is mandatory, interestingly not for NB formation, but for PML/RARα degradation [91,93]. It is proved that the PML SUMOylation at the K160 site is a crucial event for leukemogenesis and disease progression, while its inhibition prevents leukemia cells from differentiating and gaining immortal properties, further preventing proteasomal degradation [94]. Interestingly, hematopoietic progenitor cells in mouse models presented a limited growth period when transduced with SUMO-deficient K160R PML-RARα, and they finally presented myeloid hyperplasia with no progression to APL, confirming that leukemia cells’ proliferation and differentiation are significantly associated with SUMO expression [95].

**Figure 2:**
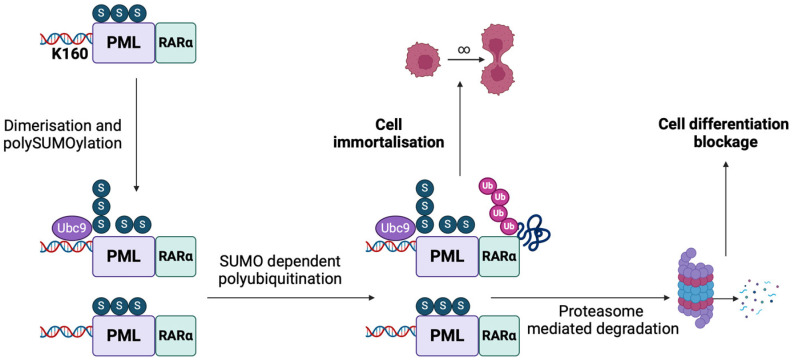
Role of PML-RARα SUMOylation in K160, which is considered mandatory for cell differentiation blockage. Cycles of polySUMOylation and polyubiquitination gradually lead to cell immortalization, being implicated in APL tumorigenic properties. [PML-RARα: promyelocytic leukemia retinoic acid receptor α protein; SUMO: small ubiquitin-related modifier; Ubc9: E2 SUMO-conjugating protein.] (The image was created using BioRender software version 04, License #TB27W7DNGU).

SUMOylation involvement in APL regarding the inhibition of cell differentiation and the enhancement of self-renewal capacity and immortal properties is illustrated in Figure 2.

Regarding the therapeutic properties of SUMOylation in APL, several studies highlight its implications in established treatment options. To begin with, As_2_O_3_, which belongs to the therapeutic choices for APL, binds directly to PML/RARα and PML, inducing their polymerization by oxidating cysteine regions and creating disulfide bonds [96]. Hyper-SUMOylation of PML/RARα results in the recruitment of the SUMO-dependent ubiquitin ligase RNF4 peptides to the proteasome, and, gradually, in the total degradation of the chimeric protein, being a promising indicator of treatment sensitivity [31,97]. Specifically, this cascade leads to RARα signaling reactivation and to NB regeneration, activating the p53 pathway and inducing APL cell apoptosis [98]. Furthermore, it has been proved that the “gold standard” of APL treatment, ATRA and ATO, may trigger SUMOylation via several mechanisms, thus inducing PML-RARα degradation [97,99,100]. These mechanisms include (i) recruitment of ubiquitin E3 ligase ring finger protein 4 (RNF4), and (ii) SENP1-mediated interchange of SUMO1 to SUMO2 at the Lys65 region of the PML peptide. The aforementioned mechanisms synergistically result in poly-ubiquitination and proteasomal degradation of PML-RARα in NBs, leading to increased apoptosis of APL cells [97,101]. Overall, this evidence combined indicates that SUMOylation is a major regulator of both APL leukemogenesis and chemosensitivity; thus, SUMO modifications could be investigated as a potential indicator of patients’ response to treatment in order to detect the small percentage of patients with refractory disease early. However, these hypotheses need to be confirmed by large in vivo clinical trials.

### 3.2. SUMO in Non-APL Acute Leukemia

Despite the revolutionary approach with ATRA-ATO in APL AML and its significant improvements in patients’ survival and quality of life, persistent obstacles remain in the development of therapeutic options with durable responses in patients with non-APL AML. Recent evidence supports the potential role of the SUMO signaling cascade in non-APL AML in terms of disease development and therapeutic evaluation. Firstly, C/EBPα, which regulates early myeloid differentiation, is reported to present one or multiple mutations in approximately 10% of AMLs, being associated with mutations in transcription factors (e.g., GATA2) or with the production of pathological p30 variants and being negatively correlated with anti-tumor effects of the p42 C/EBPα isoform’s activity [102]. Ubc9, except for being a main component of the SUMO pathway, is also a negative activator of p42 C/EBPα, decreasing its pro-differentiative capacity and being correlated with the worst disease prognosis [103,104]. Moreover, SUMO expression is positively associated with proteins of TME metabolic reprogramming in AML, mainly with insulin growth factor-like receptor 1 (IGF-1R) [105]. It is of note that inhibiting Ubc9 or IGF-1R SUMOylation sites has been reported to decrease AML severity without dysregulating leukemia cell apoptosis [105]. Dysregulated SUMOylation of other factors, for instance, hyper-SUMOylation of sPRDM16 (at lysine 568 region), and decreased HIPK2 (R868W and N958I mutated) SUMOylation, lead to transcription errors, collectively promoting proliferation and inhibiting differentiation of AML cells [106,107,108,109].

Another important aspect of SUMO involvement in the pathogenesis of non-APL AML is the regulation of DNA damage response of leukemia cells via nucleophosmin (NPM), which is encoded by the most frequent mutated gene (NPM) in non-APL AML, and human coilin-interacting nuclear ATPase protein (hCINAP) [110,111,112]. The formation of double-strand breaks subsequently leads to NPM SUMOylation inducing SUMO interactions with the tumor suppressor genes BRCA1 and BRCA2 [110,111,112]. DNA damage response progress comes along with hCINAP transportation to damage sites, and its binding with SENP3, in order to induce SENP3-mediated deSUMOylation of NPM, leading to prevention of excessive repair [110,113]. Based on these pathophysiological mechanisms, it is considered that hCINAP expression is negatively correlated with non-APL AML prognosis [110,113]. Pre-clinical in vitro studies in xenograft AML mouse models revealed higher chemosensitivity, increased levels of DDR, and induced cell apoptosis, and as a result, induced AML disease progression. Overall, the expression of hCINAP protein is considered a regulator of DNA damage response by SUMOylation of NPM, suggesting hCINAP as a promising treatment option for non-APL AML patients with poor prognosis [110,113].

SUMO implications in non-APL AML chemosensitivity represent a research area gaining increased scientific interest lately. Given that the inhibition of the SUMO cascade, acts as a regulator of several genes involved in cell proliferation, differentiation, and apoptosis in non-APL AML, targeting SUMOylation could increase non-APL AML patients’ response rates to the currently established chemotherapeutic schemas [41]. In vitro studies using AML cell lines have recently tested the potential role of deSUMOylation in chemosensitive cells after chemotherapy induction with the standard non-APL AML schema, consisting of daunorubicin, cytarabine, and etoposide [114]. Results from these studies showed that (i) ROS are the main regulator of de-SUMOylation being involved in the cellular pro-apoptotic processes, increasing the expression levels of the DDIT3 (CHOP-10 or GADD153) gene, (ii) chemoresistant leukemia cells do not induce the ROS/SUMOylation axis [114]. Additionally, SUMO has been investigated in terms of leukemic cell response to epigenetic drugs, specifically HDAC inhibitors (HDACi), via the inducement of the SUMO-2/3 modification of the chromobox protein homolog 2 (CBX2), which resulted in RNF4 recruitment and proteasome degradation finally downregulating proliferation of the AML cells [115].

## 4. Targeting SUMOylation in AML: Ongoing Clinical Trials and Pre-Clinical Studies

As previously described, targeting SUMOylation is currently considered a promising therapeutic approach for several types of advanced hematologic neoplasms, including AML. This observation has been initially proved by studying the pharmaceutical inhibition of specific sites of SUMOylation in vitro via the use of the SUMO inhibitors termed anacardic acid and 2D-08, which were proven to facilitate the ATRA-induced expression of the main gene regulators of cell survival and death in non-APL AML cells [116]. As previously mentioned herein, in AML cells showing minor or no response to treatment, chemotherapy induction did not activate the ROS/SUMO axis; this observation created the need for axis reactivation via the pharmaceutical inhibition of the SUMO pathway [41]. Anacardic acid was successfully used in this direction, confirming the hypotheses of previous studies [116]. It is worth noting that anacardic acid presents high efficacy in killing leukemic stem cells (specifically CD34^+^ CD38^low/-^CD123^+^ cells) and in limiting the growth of chemoresistant human AML cell lines xenografted to immunodeficient mouse models [41]. Some years later came the induction of 2-D08, an inhibitor of the SUMO E2 component, which was proved to enhance ROS-mediated cell death of several AML cell lines by deSUMOylating the NADPH oxidase Nox2 [117]. Both the aforementioned targets (anacardic acid, 2D-08) were used as enhancers for overcoming chemoresistance in non-APL AMLs.

After the successful use of these two primary inhibitors in vitro, follow-up pre-clinical studies proposed several new SUMO inhibitors, gradually leading to the first application in vivo of SUMOylation targeting in AML patients using the highly selective TAK-981 inhibitor [118,119,120,121,122,123,124]. TAK-981 inhibitor represents the only in vivo tested compound that directly targets SUMOylation and is suggested as the most promising for further use in clinical settings so far [118,119]. Unfortunately, the rest of the SUMO inhibitors have not yet progressed to in vivo clinical studies [116,117,124]. The currently published investigation on SUMOylation targeting in AML is summarized in Table 1.

TAK-981, termed Subasumstat, is a first-in-class SUMOylation inhibitor with previously proven significant anti-leukemic activity in preclinical models of AML, which is currently tested in a phase 1 clinical trial (NCT03648372) in patients with metastatic solid tumors and hematologic malignancies, including 25 AML patients [118,119,125]. These studies suggest that TAK-981 successfully targets AML and blast cells in vitro and in vivo, presenting a great safety profile considering its limited toxic effects on normal hematopoietic cells [118,119,125]. Additionally, it has been shown to present a synergistic effect with 5-azacytidine (AZA), a DNA-hypomethylating agent combined with the BCL-2 inhibitor Venetoclax to treat patients with relapsed or refractory AML [22,125]. TAK-981 and AZA combination is more effective in vitro and in vivo compared to the use of each medication individually, which could partially be explained by the fact that TAK-981 facilitates the transcriptional reprogramming induced by AZA, promoting apoptosis, alteration of the cell cycle, and differentiation of the leukemic cells, as well as inducing the expression of natural killer-activating ligands (MICA/B) and adhesion of AML molecules (ICAM-1) [125]. Thus, targeting the SUMO signaling pathway in advanced AML with TAK-981 and AZA combination represents a promising strategy to sensitize AML cells to AZA and reduce their immune-escape capacity. ML-792, the “precursor” of TAK-981, had been tested as a potential SUMO inhibitor based on its ability to inhibit proliferation in AML cell lines, especially in those overexpressing the Myc oncogene; however, it led to chromosome-segregation defects, further resulting in proliferation arrest and death of both AML cells and normal hematopoietic cells [124].

The results of these studies provide strong evidence for SUMOylation as a promising novel targeting pathway for AML; however, further validation of the above findings is needed by in vitro, ex vivo, and in vivo investigation, as well as by integrated bioinformatic screening. Interestingly, another finding of bioinformatics analysis of 13 frequent PTMs was that SUMOylation could be involved in the clonal evolution of neoplastic cells, being a promising therapeutic target; however, its exact role in the selection of clonal diversity within a malignancy during and after treatment has not been defined yet [126]. The selection of clonal diversity is an intrinsic heritable property being regulated by genetic and other (e.g., antigen presentation) factors. Based on a study by Fennel et al. in mouse models, single-cell epigenetic profiling could be useful, probably not in studying clonal dominance during the AML treatment process, but in identifying the clonal dominance early at diagnosis, which is associated with the rapid expansion of leukemic cells and advanced AML phenotypes [127]. Regarding SUMO inhibitors’ safety profile, current real-world data are limited to the in vitro TAK-981 study, which reported a good toxicity property of up to 40 mg/kg in mouse models [118,119]. These favorable toxicity data should prompt the conduction of follow-up studies for SUMO inhibitors’ optimal combination use with other chemotherapeutic drugs and for the transitions of these schemas to clinical trials in large AML cohorts.

## 5. Discussion and Future Perspectives

PTMs have been an area of extensive research during the last decade, and, as a result, increasing evidence regarding activator mechanisms of specific pathways, which contribute to the regulation of a wide variety of cellular processes, has emerged. Recent studies support the potential role of SUMO proteins’ participation in cell proliferation, cell division, chromosomal rearrangements, apoptotic pathways, and stress response in various types of malignancies [41]. Hematologic malignancies, including AML, encompass a heterogeneous array of neoplasms and a significant clinical challenge, mainly due to their propensity for treatment resistance and significant relapse rates in all age groups, which necessitate tailored therapeutic approaches [128,129,130]. Based on the influence of the SUMO signaling cascade on the pathogenesis of AML, further development of small molecules that specifically modulate SUMO alterations is a highly promising approach to novel targeted therapeutic evaluation for AML patients. The introduction of highly selective SUMO inhibitors represents a significant innovation in the field of SUMOylation; however, as previously described, the majority of novel targeted molecules still lack sufficient specificity and clinically relevant pharmacological properties, an issue needing extensive research in large patient cohorts to be overcome [131].

Considering that dysregulation of SUMOylation results in increased cell proliferation and decreased apoptotic rates in AML, further investigation into the regulatory pathways of the dynamic redistribution of SUMO signaling is essential. Currently, numerous novel SUMO target peptides have been identified and described using site-specific proteomics approaches [132]. More global testing of overall SUMO expression levels in each type of malignancy, for example, AML itself, should be performed in order to identify a distinct “epigenetic signature”, differentiating each neoplasm from other types of malignancy [133]. Despite the heterogeneity of epigenetic profiles in the different stages of cancer, the introduction of the idea of epigenetic fingerprints has been studied in some types of advanced tumors, with neuroblastoma being a characteristic example; various methylation sites (promoters, intragenic and intergenic regions) are a part of the tumor unique “epigenetic signature” and currently are being investigated as potential targets in clinical studies [134]. Continuous research on the power of sensitive analytical methods (e.g., mass spectrometry) and bioinformatic analyses could increase the sensitivity and specificity of PTM-specific antibodies, allowing (i) further studies of the different epigenetic profiles in the different disease stages, (ii) deeper analyses of their similarities and potential targeting, and (iii) identification of novel PTM sites with extremely low stoichiometry, but a possibly key role in disease severity and clinical course [135]. Moreover, despite the primary mechanisms regulating E2-mediated conjugation of SUMO substrates being recently described, details on substrate selection remain unclear, especially when it involves SUMO modification of lysine regions that are different from canonical SUMO consensus sites [30]. In addition, some binding peptides present high specificity towards a distinct SUMO isoform, but these preferences are particularly slight and the exact molecular basis for SUMO isoform specificity has not been analytically described yet [136]. Thus, it is considered of great importance to continue the efforts of clarifying potential mechanisms that regulate substrate specificity and SUMO isoform selection, especially in highly diverse malignancies, including AML [136].

It has yet to be clarified that hyper-SUMOylation enhances leukemia cells’ survival; thus, developing SUMOylation or de-SUMOylation inhibitors with high specificity to AML cells, whilst balancing their tolerance by normal cells, could be potentially beneficial [134]. Since leukemia cells may be partially dependent on epigenetic signaling cascades, it would be of great importance to target SUMOylation as an attractive therapeutic option to improve AML treatment. Regarding further research into SUMOylation targeting, as previously analyzed herein, PML-NBs represent promising SUMOylation targets that provide scaffolds for AML therapeutic intervention [137]. However, currently, there is no available targeting method against certain peptides to PML-NBs, and, as a result, a robust and generalized induction of PML-NB biogenesis using ATO treatment is used, which is being tested as a part of AML patients’ therapeutic schemas [138]. The induced PML-NB formation may pathologically activate the hyper-SUMOylated proteins, leading to distinct translational processes, such as protein degradation, sequestration into the PML-NBs, and dysregulated activity and modification; therefore, targeting SUMO-mediated oncoprotein oligomerization could be a potential part of the therapeutic strategy against refractory AML [138].

Finally, considering the promising results of the emerging ubiquitylation-based therapeutic technology termed ’proteolysis-targeting chimera’, which degrades the targeted substrate via the ubiquitin proteasomal system, SUMOylation may be considered a target for a selected substrate to differentiate this substrate’s peptide stability, solubility, localization, and protein–network interactions [139]. Towards this direction, a chimeric formula consisting of (i) a protein domain with high specificity in binding to the target substrate, and (ii) a SUMO domain component binding to this protein domain by a fusion linker peptide, could be taken into consideration for further research [140]. The identification of novel SUMO substrates is crucial for uncovering the functional mechanisms by which diverse epigenetic cascades interact at a cellular level to drive tumorigenic progression during cell proliferation. Although many aspects of SUMOylation remain underexplored, this post-translational modification stands out as one of the most prevalent in AML and is increasingly recognized for its role in disease pathogenesis. Based on the findings discussed, the SUMO signaling pathway holds significant potential as both a diagnostic and therapeutic target for patients with advanced or refractory AML. Its ability to enable highly specific substrate targeting, combined with advancements in high-throughput proteomics, offers a promising avenue for precision medicine in AML management.

## 6. Conclusions

AML is a highly heterogeneous hematologic malignancy, with long-term survival remaining rare for advanced disease without the use of allogeneic hematopoietic stem cell transplantation. Emerging therapeutic strategies targeting epigenetic alterations offer significant promise; however, their clinical translation is hindered by various challenges. Among these, targeting SUMOylation has emerged as a promising avenue within the realm of epigenetic therapeutics. This approach is gaining traction in post-translational AML research, paving the way for precision medicine strategies in AML treatment. Nevertheless, the complexity and diversity of SUMO-related epigenetic alterations in AML, combined with therapy-related coexisting PTMs, underscore the need for robust, multidisciplinary research. Comprehensive preclinical and clinical studies are essential to advance these novel approaches and optimize their integration into clinical practice.

## Figures and Tables

**Table 1:** Inhibitors of SUMO signaling cascade in AML patients or in vitro models.

Reference	Inhibitor Name	Use	Target	Number of Patients Enrolled
[116]	Anacardic acid	In vitro	SAE1/SAE2	N/A
[117]	2D-08	In vitro	UBC09	N/A
[118,119]	TAK-981	In vivo and in vitro	SAE1/SAE2	25 patients
[120]	ΜL-792	In vitro	SAE1/SAE2	N/A

## References

[1] Gibney E.R., Nolan C.M. (2010). Epigenetics and Gene Expression. Heredity.

[2] Satam H., Joshi K., Mangrolia U., Waghoo S., Zaidi G., Rawool S., Thakare R.P., Banday S., Mishra A.K., Das G. (2023). Next-Generation Sequencing Technology: Current Trends and Advancements. Biology.

[3] Tuscher J.J., Day J.J. (2019). Multigenerational Epigenetic Inheritance: One Step Forward, Two Generations Back. Neurobiol. Dis..

[4] Yuan M., Yang B., Rothschild G., Mann J.J., Sanford L.D., Tang X., Huang C., Wang C., Zhang W. (2023). Epigenetic Regulation in Major Depression and Other Stress-Related Disorders: Molecular Mechanisms, Clinical Relevance and Therapeutic Potential. Signal Transduct. Target. Ther..

[5] Zafar S., Fatima S.I., Schmitz M., Zerr I. (2024). Current Technologies Unraveling the Significance of Post-Translational Modifications (PTMs) as Crucial Players in Neurodegeneration. Biomolecules.

[6] Marshall C.J. (1993). Protein Prenylation: A Mediator of Protein-Protein Interactions. Science.

[7] Del Monte F., Agnetti G. (2014). Protein Post-translational Modifications and Misfolding: New Concepts in Heart Failure. PROTEOMICS–Clinical Appl..

[8] Audagnotto M., Dal Peraro M. (2017). Protein Post-Translational Modifications: In Silico Prediction Tools and Molecular Modeling. Comput. Struct. Biotechnol. J..

[9] Huang C.-H., Yang T.-T., Lin K.-I. (2024). Mechanisms and Functions of SUMOylation in Health and Disease: A Review Focusing on Immune Cells. J. Biomed. Sci..

[10] Ma X.-N., Li M.-Y., Qi G.-Q., Wei L.-N., Zhang D.-K. (2024). SUMOylation at the Crossroads of Gut Health: Insights into Physiology and Pathology. Cell Commun. Signal..

[11] Papadakos S.P., Chatzikalil E., Vakadaris G., Reppas L., Arvanitakis K., Koufakis T., Siakavellas S.I., Manolakopoulos S., Germanidis G., Theocharis S. (2024). Exploring the Role of GITR/GITRL Signaling: From Liver Disease to Hepatocellular Carcinoma. Cancers.

[12] Arvanitakis K., Papadakos S.P., Vakadaris G., Chatzikalil E., Stergiou I.E., Kalopitas G., Theocharis S., Germanidis G. (2024). Shedding Light on the Role of LAG-3 in Hepatocellular Carcinoma: Unraveling Immunomodulatory Pathways. Hepatoma Res..

[13] Papadakos S.P., Chatzikalil E., Arvanitakis K., Vakadaris G., Stergiou I.E., Koutsompina M.-L., Argyrou A., Lekakis V., Konstantinidis I., Germanidis G. (2024). Understanding the Role of Connexins in Hepatocellular Carcinoma: Molecular and Prognostic Implications. Cancers.

[14] Sahin U., de Thé H., Lallemand-Breitenbach V. (2022). Sumoylation in Physiology, Pathology and Therapy. Cells.

[15] Yuan H., Lu Y., Chan Y.-T., Zhang C., Wang N., Feng Y. (2021). The Role of Protein SUMOylation in Human Hepatocellular Carcinoma: A Potential Target of New Drug Discovery and Development. Cancers.

[16] Wemyss C., Jones E., Stentz R., Carding S.R. (2024). Acute Myeloid Leukaemia and Acute Lymphoblastic Leukaemia Classification and Metabolic Characteristics for Informing and Advancing Treatment. Cancers.

[17] Döhner H., Wei A.H., Appelbaum F.R., Craddock C., DiNardo C.D., Dombret H., Ebert B.L., Fenaux P., Godley L.A., Hasserjian R.P. (2022). Diagnosis and Management of AML in Adults: 2022 Recommendations from an International Expert Panel on Behalf of the ELN. Blood.

[18] Boscaro E., Urbino I., Catania F.M., Arrigo G., Secreto C., Olivi M., D’Ardia S., Frairia C., Giai V., Freilone R. (2023). Modern Risk Stratification of Acute Myeloid Leukemia in 2023: Integrating Established and Emerging Prognostic Factors. Cancers.

[19] Bono R., Sapienza G., Tringali S., Rotolo C., Patti C., Mulè A., Calafiore V., Santoro A., Castagna L. (2024). Allogeneic Stem Cell Transplantation in Refractory Acute Myeloid Leukaemia. Cells.

[20] Canichella M., de Fabritiis P. (2024). Cell-Based Treatment in Acute Myeloid Leukemia Relapsed after Allogeneic Stem Cell Transplantation. Biomedicines.

[21] Delaporta P., Chatzikalil E., Ladis V., Moraki M., Kattamis A. (2023). Evolving Changes in the Characteristics of Death in Transfusion Dependent Thalassemia in Greece. Blood.

[22] Chatzikalil E., Roka K., Diamantopoulos P.T., Rigatou E., Avgerinou G., Kattamis A., Solomou E.E. (2024). Venetoclax Combination Treatment of Acute Myeloid Leukemia in Adolescents and Young Adult Patients. J. Clin. Med..

[23] Totiger T.M., Ghoshal A., Zabroski J., Sondhi A., Bucha S., Jahn J., Feng Y., Taylor J. (2023). Targeted Therapy Development in Acute Myeloid Leukemia. Biomedicines.

[24] Baylin S.B., Fearon E.R., Vogelstein B., de Bustros A., Sharkis S.J., Burke P.J., Staal S.P., Nelkin B.D. (1987). Hypermethylation of the 5’region of the Calcitonin Gene Is a Property of Human Lymphoid and Acute Myeloid Malignancies. Blood.

[25] Pfeifer G.P., Steigerwald S., Boehm T.L.J., Drahovsky D. (1988). DNA Methylation Levels in Acute Human Leukemia. Cancer Lett..

[26] Celen A.B., Sahin U. (2020). Sumoylation on Its 25th Anniversary: Mechanisms, Pathology, and Emerging Concepts. FEBS J..

[27] Guo D., Li M., Zhang Y., Yang P., Eckenrode S., Hopkins D., Zheng W., Purohit S., Podolsky R.H., Muir A. (2004). A Functional Variant of SUMO4, a New IκBα Modifier, Is Associated with Type 1 Diabetes. Nat. Genet..

[28] Bohren K.M., Nadkarni V., Song J.H., Gabbay K.H., Owerbach D. (2004). A M55V Polymorphism in a Novel SUMO Gene (SUMO-4) Differentially Activates Heat Shock Transcription Factors and Is Associated with Susceptibility to Type I Diabetes Mellitus. J. Biol. Chem..

[29] Wang L., Wansleeben C., Zhao S., Miao P., Paschen W., Yang W. (2014). SUMO 2 Is Essential While SUMO 3 Is Dispensable for Mouse Embryonic Development. EMBO Rep..

[30] Gareau J.R., Lima C.D. (2010). The SUMO Pathway: Emerging Mechanisms That Shape Specificity, Conjugation and Recognition. Nat. Rev. Mol. cell Biol..

[31] Tatham M.H., Jaffray E., Vaughan O.A., Desterro J.M.P., Botting C.H., Naismith J.H., Hay R.T. (2001). Polymeric Chains of SUMO-2 and SUMO-3 Are Conjugated to Protein Substrates by SAE1/SAE2 and Ubc9. J. Biol. Chem..

[32] Hendriks I.A., D’souza R.C.J., Yang B., Verlaan-de Vries M., Mann M., Vertegaal A.C.O. (2014). Uncovering Global SUMOylation Signaling Networks in a Site-Specific Manner. Nat. Struct. Mol. Biol..

[33] Sundvall M. (2020). Role of Ubiquitin and SUMO in Intracellular Trafficking. Curr. Issues Mol. Biol..

[34] Vijay-Kumar S., Bugg C.E., Cook W.J. (1987). Structure of Ubiquitin Refined at 1.8 Åresolution. J. Mol. Biol..

[35] Bayer P., Arndt A., Metzger S., Mahajan R., Melchior F., Jaenicke R., Becker J. (1998). Structure Determination of the Small Ubiquitin-Related Modifier SUMO-1. J. Mol. Biol..

[36] Pichler A., Fatouros C., Lee H., Eisenhardt N. (2017). SUMO Conjugation–a Mechanistic View. Biomol. Concepts.

[37] Hutten S., Chachami G., Winter U., Melchior F., Lamond A.I. (2014). A Role for the CB-Associated SUMO Isopeptidase USPL1 in RNAPII-Mediated SnRNA Transcription. J. Cell Sci..

[38] Werner A., Flotho A., Melchior F. (2012). The RanBP2/RanGAP1∗ SUMO1/Ubc9 Complex Is a Multisubunit SUMO E3 Ligase. Mol. Cell.

[39] Eisenhardt N., Chaugule V.K., Koidl S., Droescher M., Dogan E., Rettich J., Sutinen P., Imanishi S.Y., Hofmann K., Palvimo J.J. (2015). A New Vertebrate SUMO Enzyme Family Reveals Insights into SUMO-Chain Assembly. Nat. Struct. Mol. Biol..

[40] Cappadocia L., Pichler A., Lima C.D. (2015). Structural Basis for Catalytic Activation by the Human ZNF451 SUMO E3 Ligase. Nat. Struct. Mol. Biol..

[41] Boulanger M., Paolillo R., Piechaczyk M., Bossis G. (2019). The SUMO Pathway in Hematomalignancies and Their Response to Therapies. Int. J. Mol. Sci..

[42] Stankovic-Valentin N., Melchior F. (2018). Control of SUMO and Ubiquitin by ROS: Signaling and Disease Implications. Mol. Aspects Med..

[43] Seifert A., Schofield P., Barton G.J., Hay R.T. (2015). Proteotoxic Stress Reprograms the Chromatin Landscape of SUMO Modification. Sci. Signal..

[44] Gervais C., Dano L., Perrusson N., Helias C., Jeandidier E., Galoisy A.C., Ittel A., Herbrecht R., Bilger K., Mauvieux L. (2013). A Translocation t (2; 8)(Q12; P11) Fuses FGFR1 to a Novel Partner Gene, RANBP2/NUP358, in a Myeloproliferative/Myelodysplastic Neoplasm. Leukemia.

[45] Lee S.E., Kang S.Y., Takeuchi K., Ko Y.H. (2014). Identification of RANBP2–ALK Fusion in ALK Positive Diffuse Large B-cell Lymphoma. Hematol. Oncol..

[46] Lim J.-H., Jang S., Park C.-J., Cho Y.-U., Lee J.-H., Lee K.-H., Lee J.-O., Shin J.-Y., Kim J.-I., Huh J. (2014). RANBP2-ALK Fusion Combined with Monosomy 7 in Acute Myelomonocytic Leukemia. Cancer Genet..

[47] Bettermann K., Benesch M., Weis S., Haybaeck J. (2012). SUMOylation in Carcinogenesis. Cancer Lett..

[48] Zhao Q., Ma Y., Li Z., Zhang K., Zheng M., Zhang S. (2020). The Function of SUMOylation and Its Role in the Development of Cancer Cells under Stress Conditions: A Systematic Review. Stem Cells Int..

[49] Lee J., Chu I., Heo J., Calvisi D.F., Sun Z., Roskams T., Durnez A., Demetris A.J., Thorgeirsson S.S. (2004). Classification and Prediction of Survival in Hepatocellular Carcinoma by Gene Expression Profiling. Hepatology.

[50] Chen X.-L., Wang S.-F., Liang X.-T., Liang H.-X., Wang T.-T., Wu S.-Q., Qiu Z.-J., Zhan R., Xu Z.-S. (2018). SENP2 Exerts an Anti-Tumor Effect on Chronic Lymphocytic Leukemia Cells through the Inhibition of the Notch and NF-ΚB Signaling Pathways. Int. J. Oncol..

[51] Taheri M., Oskooei V.K., Ghafouri-Fard S. (2019). Protein Inhibitor of Activated STAT Genes Are Differentially Expressed in Breast Tumor Tissues. Per. Med..

[52] Tuccilli C., Baldini E., Sorrenti S., Di Gioia C., Bosco D., Ascoli V., Mian C., Barollo S., Rendina R., Coccaro C. (2015). PAPILLARY THYROID CANCER IS CHARACTERIZED BY ALTERED EXPRESSION OF GENES INVOLVED IN THE SUMOYLATION PROCESS. J. Biol. Regul. Homeost. Agents.

[53] Qian J., Luo Y., Gu X., Wang X. (2013). Inhibition of SENP6-Induced Radiosensitization of Human Hepatocellular Carcinoma Cells by Blocking Radiation-Induced NF-ΚB Activation. Cancer Biother. Radiopharm..

[54] Stefanska B., Cheishvili D., Suderman M., Arakelian A., Huang J., Hallett M., Han Z.-G., Al-Mahtab M., Akbar S.M.F., Khan W.A. (2014). Genome-Wide Study of Hypomethylated and Induced Genes in Patients with Liver Cancer Unravels Novel Anticancer Targets. Clin. Cancer Res..

[55] Zhao Z., Tan X., Zhao A., Zhu L., Yin B., Yuan J., Qiang B., Peng X. (2012). MicroRNA-214-Mediated UBC9 Expression in Glioma. BMB Rep..

[56] Yang H., Tang Y., Guo W., Du Y., Wang Y., Li P., Zang W., Yin X., Wang H., Chu H. (2014). Up-Regulation of MicroRNA-138 Induce Radiosensitization in Lung Cancer Cells. Tumor Biol..

[57] Zheng C., Li J., Wang Q., Liu W., Zhou J., Liu R., Zeng Q., Peng X., Huang C., Cao P. (2015). MicroRNA-195 Functions as a Tumor Suppressor by Inhibiting CBX4 in Hepatocellular Carcinoma Retraction in/10.3892/or. 2021.8145. Oncol. Rep..

[58] Wang C., Tao W., Ni S., Chen Q., Zhao Z., Ma L., Fu Y., Jiao Z. (2015). Tumor-suppressive Micro RNA-145 Induces Growth Arrest by Targeting SENP 1 in Human Prostate Cancer Cells. Cancer Sci..

[59] Jiang M., Zhang W., Zhang R., Liu P., Ye Y., Yu W., Guo X., Yu J. (2020). Cancer Exosome-Derived MiR-9 and MiR-181a Promote the Development of Early-Stage MDSCs via Interfering with SOCS3 and PIAS3 Respectively in Breast Cancer. Oncogene.

[60] Giraldo N.A., Sanchez-Salas R., Peske J.D., Vano Y., Becht E., Petitprez F., Validire P., Ingels A., Cathelineau X., Fridman W.H. (2019). The Clinical Role of the TME in Solid Cancer. Br. J. Cancer.

[61] Mastrogeorgiou M., Chatzikalil E., Theocharis S., Papoudou-Bai A., Péoc’h M., Mobarki M., Karpathiou G. (2024). The Immune Microenvironment of Cancer of the Uterine Cervix. Histol. Histopathol..

[62] You M., Xie Z., Zhang N., Zhang Y., Xiao D., Liu S., Zhuang W., Li L., Tao Y. (2023). Signaling Pathways in Cancer Metabolism: Mechanisms and Therapeutic Targets. Signal Transduct. Target. Ther..

[63] Hannoun Z., Maarifi G., Chelbi-Alix M.K. (2016). The Implication of SUMO in Intrinsic and Innate Immunity. Cytokine Growth Factor Rev..

[64] Desterro J.M.P., Rodriguez M.S., Hay R.T. (1998). SUMO-1 Modification of IκBα Inhibits NF-ΚB Activation. Mol. Cell.

[65] Chanda A., Sarkar A., Bonni S. (2018). The SUMO System and TGFβ Signaling Interplay in Regulation of Epithelial-Mesenchymal Transition: Implications for Cancer Progression. Cancers.

[66] Xu H., Wang H., Zhao W., Fu S., Li Y., Ni W., Xin Y., Li W., Yang C., Bai Y. (2020). SUMO1 Modification of Methyltransferase-like 3 Promotes Tumor Progression via Regulating Snail MRNA Homeostasis in Hepatocellular Carcinoma. Theranostics.

[67] Gu Z., Chen X., Yang W., Qi Y., Yu H., Wang X., Gong Y., Chen Q., Zhong B., Dai L. (2021). The SUMOylation of TAB2 Mediated by TRIM60 Inhibits MAPK/NF-ΚB Activation and the Innate Immune Response. Cell. Mol. Immunol..

[68] Zheng X., Wang C., Lin W., Lin C., Han D., Xie Q., Lai J., Yang C. (2022). Importation of Chloroplast Proteins under Heat Stress Is Facilitated by Their SUMO Conjugations. New Phytol..

[69] Wang L., Ma Q., Yang W., Mackensen G.B., Paschen W. (2012). Moderate Hypothermia Induces Marked Increase in Levels and Nuclear Accumulation of SUMO 2/3-conjugated Proteins in Neurons. J. Neurochem..

[70] Keiten-Schmitz J., Wagner K., Piller T., Kaulich M., Alberti S., Müller S. (2020). The Nuclear SUMO-Targeted Ubiquitin Quality Control Network Regulates the Dynamics of Cytoplasmic Stress Granules. Mol. Cell.

[71] Ferdaoussi M., Dai X., Jensen M.V., Wang R., Peterson B.S., Huang C., Ilkayeva O., Smith N., Miller N., Hajmrle C. (2015). Isocitrate-to-SENP1 Signaling Amplifies Insulin Secretion and Rescues Dysfunctional β Cells. J. Clin. Investig..

[72] Shao L., Zhou H.J., Zhang H., Qin L., Hwa J., Yun Z., Ji W., Min W. (2015). SENP1-Mediated NEMO DeSUMOylation in Adipocytes Limits Inflammatory Responses and Type-1 Diabetes Progression. Nat. Commun..

[73] Corces M.R., Buenrostro J.D., Wu B., Greenside P.G., Chan S.M., Koenig J.L., Snyder M.P., Pritchard J.K., Kundaje A., Greenleaf W.J. (2016). Lineage-Specific and Single-Cell Chromatin Accessibility Charts Human Hematopoiesis and Leukemia Evolution. Nat. Genet..

[74] Eriksson A., Lennartsson A., Lehmann S. (2015). Epigenetic Aberrations in Acute Myeloid Leukemia: Early Key Events during Leukemogenesis. Exp. Hematol..

[75] Goldman S.L., Hassan C., Khunte M., Soldatenko A., Jong Y., Afshinnekoo E., Mason C.E. (2019). Epigenetic Modifications in Acute Myeloid Leukemia: Prognosis, Treatment, and Heterogeneity. Front. Genet..

[76] Rodrigues C.P., Shvedunova M., Akhtar A. (2021). Epigenetic Regulators as the Gatekeepers of Hematopoiesis. Trends Genet..

[77] Lo-Coco F., Avvisati G., Vignetti M., Thiede C., Orlando S.M., Iacobelli S., Ferrara F., Fazi P., Cicconi L., Di Bona E. (2013). Retinoic Acid and Arsenic Trioxide for Acute Promyelocytic Leukemia. N. Engl. J. Med..

[78] Avgerinou G., Solomou E., Filippidou M., Perganti F., Roka K., Rigatou E., Katsibardi K., Glentis S., Vlachou A., Binenbaum I. (2024). Chemotherapy-Free Approach with Arsenic Trioxide and All-Trans Retinoic Acid in Children with Acute Promyelocytic Leukemia. Leuk. Lymphoma.

[79] Stahl M., Tallman M.S. (2019). Acute Promyelocytic Leukemia (APL): Remaining Challenges towards a Cure for All. Leuk. Lymphoma.

[80] Huang D.-P., Yang L.-C., Chen Y.-Q., Wan W.-Q., Zhou D.-H., Mai H.-R., Li W.-L., Yang L.-H., Lan H.-K., Chen H.-Q. (2023). Long-Term Outcome of Children with Acute Promyelocytic Leukemia: A Randomized Study of Oral versus Intravenous Arsenic by SCCLG-APL Group. Blood Cancer J..

[81] Ma Y.-F., Lu Y., Wu Q., Lou Y.-J., Yang M., Xu J.-Y., Sun C.-H., Mao L.-P., Xu G.-X., Li L. (2022). Oral Arsenic and Retinoic Acid for High-Risk Acute Promyelocytic Leukemia. J. Hematol. Oncol..

[82] Ravandi F., Koumenis I., Johri A., Tallman M., Roboz G.J., Strickland S., Garcia-Manero G., Borthakur G., Naqvi K., Meyer M. (2019). Oral Arsenic Trioxide ORH-2014 Pharmacokinetic and Safety Profile in Patients with Advanced Hematologic Disorders. Haematologica.

[83] Wang L., Qian J., Yang Y., Gu C. (2021). Novel Insights into the Impact of the SUMOylation Pathway in Hematological Malignancies (Review). Int. J. Oncol..

[84] de Thé H., Lavau C., Marchio A., Chomienne C., Degos L., Dejean A. (1991). The PML-RARα Fusion MRNA Generated by the t (15; 17) Translocation in Acute Promyelocytic Leukemia Encodes a Functionally Altered RAR. Cell.

[85] Boddy M.N., Howe K., Etkin L.D., Solomon E., Freemont P.S. (1996). PIC 1, a Novel Ubiquitin-like Protein Which Interacts with the PML Component of a Multiprotein Complex That Is Disrupted in Acute Promyelocytic Leukaemia. Oncogene.

[86] Weis K., Rambaud S., Lavau C., Jansen J., Carvalho T., Carmo-Fonseca M., Lamond A., Dejean A. (1994). Retinoic Acid Regulates Aberrant Nuclear Localization of PML-RARα in Acute Promyelocytic Leukemia Cells. Cell.

[87] Koken M.H., Puvion-Dutilleul F., Guillemin M.C., Viron A., Linares-Cruz G., Stuurman N., De Jong L., Szostecki C., Calvo F., Chomienne C. (1994). The t (15; 17) Translocation Alters a Nuclear Body in a Retinoic Acid-reversible Fashion. EMBO J..

[88] Daniel M.T., Koken M., Romagne O., Barbey S., Bazarbachi A., Stadler M., Guillemin M.C., Degos L., Chomienne C., de The H. (1993). PML Protein Expression in Hematopoietic and Acute Promyelocytic Leukemia Cells. Blood.

[89] Zhong S., Müller S., Ronchetti S., Freemont P.S., Dejean A., Pandolfi P.P. (2000). Role of SUMO-1–Modified PML in Nuclear Body Formation. Blood, J. Am. Soc. Hematol..

[90] Sahin U., Ferhi O., Jeanne M., Benhenda S., Berthier C., Jollivet F., Niwa-Kawakita M., Faklaris O., Setterblad N., de Thé H. (2014). Oxidative Stress–Induced Assembly of PML Nuclear Bodies Controls Sumoylation of Partner Proteins. J. Cell Biol..

[91] Shen T.H., Lin H.K., Scaglioni P.P., Yung T.M., Pandolfi P.P. (2006). The Mechanisms of PML-Nuclear Body Formation. Mol. Cell.

[92] Liang Y.-C., Lee C.-C., Yao Y.-L., Lai C.-C., Schmitz M.L., Yang W.-M. (2016). SUMO5, a Novel Poly-SUMO Isoform, Regulates PML Nuclear Bodies. Sci. Rep..

[93] Matunis M.J., Zhang X.-D., Ellis N.A. (2006). SUMO: The Glue That Binds. Dev. Cell.

[94] Zhu J., Zhou J., Peres L., Riaucoux F., Honoré N., Kogan S. (2005). A Sumoylation Site in PML/RARA Is Essential for Leukemic Transformation. Cancer Cell.

[95] Wojiski S., Guibal F.C., Kindler T., Lee B.H., Jesneck J.L., Fabian A., Tenen D.G., Gilliland D.G. (2009). PML–RARα Initiates Leukemia by Conferring Properties of Self-Renewal to Committed Promyelocytic Progenitors. Leukemia.

[96] Jeanne M., Lallemand-Breitenbach V., Ferhi O., Koken M., Le Bras M., Duffort S., Peres L., Berthier C., Soilihi H., Raught B. (2010). PML/RARA Oxidation and Arsenic Binding Initiate the Antileukemia Response of As2O3. Cancer Cell.

[97] Lallemand-Breitenbach V., Jeanne M., Benhenda S., Nasr R., Lei M., Peres L., Zhou J., Zhu J., Raught B., de Thé H. (2008). Arsenic Degrades PML or PML–RARα through a SUMO-Triggered RNF4/Ubiquitin-Mediated Pathway. Nat. Cell Biol..

[98] Ablain J., Rice K., Soilihi H., De Reynies A., Minucci S., de Thé H. (2014). Activation of a Promyelocytic Leukemia–Tumor Protein 53 Axis Underlies Acute Promyelocytic Leukemia Cure. Nat. Med..

[99] Breitman T.R., Collins S.J., Keene B.R. (1981). Terminal Differentiation of Human Promyelocytic Leukemic Cells in Primary Culture in Response to Retinoic Acid. Blood.

[100] Ablain J., Leiva M., Peres L., Fonsart J., Anthony E., de Thé H. (2013). Uncoupling RARA Transcriptional Activation and Degradation Clarifies the Bases for APL Response to Therapies. J. Exp. Med..

[101] Fasci D., Anania V.G., Lill J.R., Salvesen G.S. (2015). SUMO Deconjugation Is Required for Arsenic-Triggered Ubiquitylation of PML. Sci. Signal..

[102] Ohlsson E., Schuster M.B., Hasemann M., Porse B.T. (2016). The Multifaceted Functions of C/EBPα in Normal and Malignant Haematopoiesis. Leukemia.

[103] Geletu M., Balkhi M.Y., Peer Zada A.A., Christopeit M., Pulikkan J.A., Trivedi A.K., Tenen D.G., Behre G. (2007). Target Proteins of C/EBPαp30 in AML: C/EBPαp30 Enhances Sumoylation of C/EBPαp42 via up-Regulation of Ubc9. Blood J. Am. Soc. Hematol..

[104] Hankey W., Silver M., Sun H., Zibello T., Berliner N., Khanna-Gupta A. (2011). Differential Effects of Sumoylation on the Activities of CCAAT Enhancer Binding Protein Alpha (C/EBPα) P42 versus P30 May Contribute in Part, to Aberrant C/EBPα Activity in Acute Leukemias. Hematol. Rep..

[105] Zhang J., Huang F.-F., Wu D.-S., Li W.-J., Zhan H.-E., Peng M.-Y., Fang P., Cao P.-F., Zhang M.-M., Zeng H. (2015). SUMOylation of Insulin-like Growth Factor 1 Receptor, Promotes Proliferation in Acute Myeloid Leukemia. Cancer Lett..

[106] Dong S., Chen J. (2015). SUMOylation of SPRDM16 Promotes the Progression of Acute Myeloid Leukemia. BMC Cancer.

[107] Nishikata I., Nakahata S., Saito Y., Kaneda K., Ichihara E., Yamakawa N., Morishita K. (2011). Sumoylation of MEL1S at Lysine 568 and Its Interaction with CtBP Facilitates Its Repressor Activity and the Blockade of G-CSF-Induced Myeloid Differentiation. Oncogene.

[108] Li X.L., Arai Y., Harada H., Shima Y., Yoshida H., Rokudai S., Aikawa Y., Kimura A., Kitabayashi I. (2007). Mutations of the HIPK2 Gene in Acute Myeloid Leukemia and Myelodysplastic Syndrome Impair AML1-and P53-Mediated Transcription. Oncogene.

[109] Sæther T., Pattabiraman D.R., Alm-Kristiansen A.H., Vogt-Kielland L.T., Gonda T.J., Gabrielsen O.S. (2011). A Functional SUMO-Interacting Motif in the Transactivation Domain of c-Myb Regulates Its Myeloid Transforming Ability. Oncogene.

[110] Xu R., Yu S., Zhu D., Huang X., Xu Y., Lao Y., Tian Y., Zhang J., Tang Z., Zhang Z. (2019). HCINAP Regulates the DNA-Damage Response and Mediates the Resistance of Acute Myelocytic Leukemia Cells to Therapy. Nat. Commun..

[111] Döhner H., Weisdorf D.J., Bloomfield C.D. (2015). Acute Myeloid Leukemia. N. Engl. J. Med..

[112] Mendez L.M., Posey R.R., Pandolfi P.P. (2019). The Interplay between the Genetic and Immune Landscapes of AML: Mechanisms and Implications for Risk Stratification and Therapy. Front. Oncol..

[113] Haindl M., Harasim T., Eick D., Muller S. (2008). The Nucleolar SUMO-specific Protease SENP3 Reverses SUMO Modification of Nucleophosmin and Is Required for RRNA Processing. EMBO Rep..

[114] Bossis G., Sarry J.-E., Kifagi C., Ristic M., Saland E., Vergez F., Salem T., Boutzen H., Baik H., Brockly F. (2014). The ROS/SUMO Axis Contributes to the Response of Acute Myeloid Leukemia Cells to Chemotherapeutic Drugs. Cell Rep..

[115] Di Costanzo A., Del Gaudio N., Conte L., Dell’Aversana C., Vermeulen M., de Thé H., Migliaccio A., Nebbioso A., Altucci L. (2018). The HDAC Inhibitor SAHA Regulates CBX2 Stability via a SUMO-Triggered Ubiquitin-Mediated Pathway in Leukemia. Oncogene.

[116] Baik H., Boulanger M., Hosseini M., Kowalczyk J., Zaghdoudi S., Salem T., Sarry J.-E., Hicheri Y., Cartron G., Piechaczyk M. (2018). Targeting the SUMO Pathway Primes All-Trans Retinoic Acid–Induced Differentiation of Nonpromyelocytic Acute Myeloid Leukemias. Cancer Res..

[117] Kim Y.S., Keyser S.G.L., Schneekloth J.S. (2014). Synthesis of 2′,3′,4′-Trihydroxyflavone (2-D08), an Inhibitor of Protein Sumoylation. Bioorg. Med. Chem. Lett..

[118] Kim H.S., Kim B.-R., Dao T.T.P., Kim J.-M., Kim Y.-J., Son H., Jo S., Kim D., Kim J., Suh Y.J. (2023). TAK-981, a SUMOylation Inhibitor, Suppresses AML Growth Immune-Independently. Blood Adv..

[119] Lightcap E.S., Yu P., Grossman S., Song K., Khattar M., Xega K., He X., Gavin J.M., Imaichi H., Garnsey J.J. (2021). A Small-Molecule SUMOylation Inhibitor Activates Antitumor Immune Responses and Potentiates Immune Therapies in Preclinical Models. Sci. Transl. Med..

[120] Mohrbacher A.M., Yang A.S., Groshen S., Kummar S., Gutierrez M.E., Kang M.H., Tsao-Wei D., Reynolds C.P., Newman E.M., Maurer B.J. (2017). Phase I Study of Fenretinide Delivered Intravenously in Patients with Relapsed or Refractory Hematologic Malignancies: A California Cancer Consortium Trial. Clin. Cancer Res..

[121] Morad S.A.F., Davis T.S., Kester M., Loughran T.P., Cabot M.C. (2015). Dynamics of Ceramide Generation and Metabolism in Response to Fenretinide—Diversity within and among Leukemia. Leuk. Res..

[122] Kroonen J.S., Wouters A.K., de Graaf I.J., Remst D.F.G., Kumar S., Wachsmann T.L.A., Teunisse A.F.A.S., Roelands J.P., de Miranda N.F.C.C., Griffioen M. (2024). Targeting Epigenetic Regulation and Post-Translational Modification with 5-Aza-2’ Deoxycytidine and SUMO E1 Inhibition Augments T-Cell Receptor Therapy. J. Immunother. Cancer.

[123] Zhou P., Chen X., Li M., Tan J., Zhang Y., Yuan W., Zhou J., Wang G. (2019). 2-D08 as a SUMOylation Inhibitor Induced ROS Accumulation Mediates Apoptosis of Acute Myeloid Leukemia Cells Possibly through the DeSUMOylation of NOX2. Biochem. Biophys. Res. Commun..

[124] He X., Riceberg J., Soucy T., Koenig E., Minissale J., Gallery M., Bernard H., Yang X., Liao H., Rabino C. (2017). Probing the Roles of SUMOylation in Cancer Cell Biology by Using a Selective SAE Inhibitor. Nat. Chem. Biol..

[125] Gabellier L., De Toledo M., Chakraborty M., Akl D., Hallal R., Aqrouq M., Buonocore G., Recasens-Zorzo C., Cartron G., Delort A. (2024). SUMOylation Inhibitor TAK-981 (Subasumstat) Synergizes with 5-Azacytidine in Preclinical Models of Acute Myeloid Leukemia. Haematologica.

[126] Minguez P., Parca L., Diella F., Mende D.R., Kumar R., Helmer-Citterich M., Gavin A.C., Van Noort V., Bork P. (2012). Deciphering a Global Network of Functionally Associated Post-Translational Modifications. Mol. Syst. Biol..

[127] Fennell K.A., Vassiliadis D., Lam E.Y.N., Martelotto L.G., Balic J.J., Hollizeck S., Weber T.S., Semple T., Wang Q., Miles D.C. (2022). Non-Genetic Determinants of Malignant Clonal Fitness at Single-Cell Resolution. Nature.

[128] Chatzikalil E., Stergiou I.E., Papadakos S.P., Konstantinidis I., Theocharis S. (2024). The Clinical Relevance of the EPH/Ephrin Signaling Pathway in Pediatric Solid and Hematologic Malignancies. Int. J. Mol. Sci..

[129] Yang Y., Xia Z., Wang X., Zhao X., Sheng Z., Ye Y., He G., Zhou L., Zhu H., Xu N. (2018). Small-Molecule Inhibitors Targeting Protein SUMOylation as Novel Anticancer Compounds. Mol. Pharmacol..

[130] Wild N., Kaiser C.S., Wunderlich G., Liebau E., Wrenger C. (2024). Protein SUMOylation and Its Functional Role in Nuclear Receptor Control. Receptors.

[131] Kukkula A., Ojala V.K., Mendez L.M., Sistonen L., Elenius K., Sundvall M. (2021). Therapeutic Potential of Targeting the SUMO Pathway in Cancer. Cancers.

[132] Meulmeester E., Kunze M., Hsiao H.H., Urlaub H., Melchior F. (2008). Mechanism and Consequences for Paralog-Specific Sumoylation of Ubiquitin-Specific Protease 25. Mol. Cell.

[133] Gu Y., Fang Y., Wu X., Xu T., Hu T., Xu Y., Ma P., Wang Q., Shu Y. (2023). The Emerging Roles of SUMOylation in the Tumor Microenvironment and Therapeutic Implications. Exp. Hematol. Oncol..

[134] Gómez S., Castellano G., Mayol G., Suñol M., Queiros A., Bibikova M., Nazor K.L., Loring J.F., Lemos I., Rodríguez E. (2015). DNA Methylation Fingerprint of Neuroblastoma Reveals New Biological and Clinical Insights. Epigenomics.

[135] Dunphy K., Dowling P., Bazou D., O’Gorman P. (2021). Current Methods of Post-Translational Modification Analysis and Their Applications in Blood Cancers. Cancers.

[136] Shao X., Chen Y., Xu A., Xiang D., Wang W., Du W., Huang Y., Zhang X., Cai M., Xia Z. (2022). Deneddylation of PML/RARα Reconstructs Functional PML Nuclear Bodies via Orchestrating Phase Separation to Eradicate APL. Cell Death Differ..

[137] Voisset E., Moravcsik E., Stratford E.W., Jaye A., Palgrave C.J., Hills R.K., Salomoni P., Kogan S.C., Solomon E., Grimwade D. (2018). Pml Nuclear Body Disruption Cooperates in APL Pathogenesis and Impairs DNA Damage Repair Pathways in Mice. Blood.

[138] Li Y., Ma X., Wu W., Chen Z., Meng G. (2020). PML Nuclear Body Biogenesis, Carcinogenesis, and Targeted Therapy. Trends Cancer.

[139] Sakamoto K.M., Kim K.B., Kumagai A., Mercurio F., Crews C.M., Deshaies R.J. (2001). Protacs: Chimeric Molecules That Target Proteins to the Skp1–Cullin–F Box Complex for Ubiquitination and Degradation. Proc. Natl. Acad. Sci. USA.

[140] Chen X., Zaro J.L., Shen W.-C. (2013). Fusion Protein Linkers: Property, Design and Functionality. Adv. Drug Deliv. Rev..

